# Self-arrangement of nanoparticles toward crystalline metal oxides with high surface areas and tunable 3D mesopores

**DOI:** 10.1038/srep21496

**Published:** 2016-02-19

**Authors:** Hyung Ik Lee, Yoon Yun Lee, Dong-Uk Kang, Kirim Lee, Young-Uk Kwon, Ji Man Kim

**Affiliations:** 1 Department of Chemistry, Sungkyunkwan University, Suwon, 440-746, Republic of Korea; 2 SKKU Advanced Institute of Nanotechnology, Sungkyunkwan University, Suwon, 440-746 Korea

## Abstract

We demonstrate a new design concept where the interaction between silica nanoparticles (about 1.5 nm in diameter) with titania nanoparticles (anatase, about 4 nm or 6 nm in diameter) guides a successful formation of mesoporous titania with crystalline walls and controllable porosity. At an appropriate solution pH (~1.5, depending on the deprotonation tendencies of two types of nanoparticles), the smaller silica nanoparticles, which attach to the surface of the larger titania nanoparticles and provide a portion of inactive surface and reactive surface of titania nanoparticles, dictate the direction and the degree of condensation of the titania nanoparticles, resulting in a porous 3D framework. Further crystallization by a hydrothermal treatment and subsequent removal of silica nanoparticles result in a mesoporous titania with highly crystalline walls and tunable mesopore sizes. A simple control of the Si/Ti ratio verified the versatility of the present method through the successful control of mean pore diameter in the range of 2–35 nm and specific surface area in the ranges of 180–250 m^2^ g^−1^. The present synthesis method is successfully extended to other metal oxides, their mixed oxides and analogues with different particle sizes, regarding as a general method for mesoporous metal (or mixed metal) oxides.

Mesoporous transition metal oxides, which have crystalline framework of metal oxides as well as well-developed controllable porosity, have been considered to have extensive promising applications^1^,^2^. Particularly, many researchers have studied mesoporous metal oxides, such as titania, zirconia, niobia, tin oxide and mixed oxides, due to the inherent framework functionalities and the potential applications^3^,^4^,^5^,^6^,^7^,^8^,^9^,^10^,^11^,^12^,^13^,^14^,^15^,^16^,^17^,^18^,^19^,^20^,^21^,^22^,^23^,^24^,^25^. In order to make use of the unique physicochemical properties of metal oxides, the walls have to be highly crystalline. Highly crystalline wall of a porous material is also crucial for the high thermal and chemical stability of the porous structures.

The controllability of pore properties such as the porosity and the pore dimension is important for applications that require controlled molecular diffusion or use of molecules with various sizes and functionalites. In addition, considering future uses, the facility and scalability of the method can become an important factor to consider. There have been a lot of researches on the preparation of porous transition metal oxides^26^. The use of alcohol vapor, which is a byproduct of the reaction between a metal alkoxide with water, as the pore generating agent to produce mesoporous (or macroporous) metal oxides has been demonstrated^27^. However, the lack of the crystallinity of the framework seriously restricts the range of applications of this class of materials. Synergetic supramolecular assembly between organic surfactants (soft template) and framework precursors has been widely investigated to obtain mesoporous metal oxides^28^,^29^. This sol-gel method is capable of controlling the pore size and pore morphology. However, most of the resultant mesoporous metal oxides have amorphous framework, and the mesostructures based on the amorphous framework collapse and form thermodynamically stable non-porous solid during the surfactant removal and crystallization process at high temperature, invalidating the intended function of the surfactant. To overcome this problem, reinforcement based on rigid templates has been applied, instead of the soft organic templates. The surfactant, which has been directly removed to create mesopores^30^, or post-impregnated carbon precursor^31^ is carbonized to sustain the mesostructure during the crystallization and after the template removal process. However, the procedure is complex and requires certain specific features of the surfactants, which hardly can be extended as a generalized route. A nano-replication route^32^,^33^,^34^ using ordered mesoporous silica^35^ or carbon^32^ as a rigid template is proposed and proved to be capable of producing mesoporous metal oxides with crystalline frameworks^36^,^37^,^38^,^39^,^40^,^41^,^42^. However, this route has critical limitation in scaling-up mainly because this method involves many tedious steps including synthesis of mesoporous silica, removal of surfactant, and the infiltration of metal oxide precursors into the pores of silica that require fine controls. More importantly, this nano-replication route has pre-limited pore tunability due to the hardly controllable framework thickness of mesoporous silica template. Currently, development of a rational synthesis pathway, which overcomes the aforementioned limitations, for the crystalline metal oxides with controllable 3D mesoporosity is still remained as a prerequisite for their wide range of foreseen applications such as photovoltaics^13^,^43^,^44^, photocatalysis^45^,^46^, gas sensor^22^,^47^, rechargeable batteries^19^,^48^,^49^, and optoelectronic devices^50^.

Herein, we describe a silica nanoparticle-assisted novel design concept for mesoporous titania that have all of the above-mentioned desirable features, i.e., crystalline framework, well developed controllable 3D mesoporosity and facile process (Fig. 1). The present synthesis strategy, based on facile sol-gel synthesis in the absence of expensive organic surfactant templates, enables a systematical control of the pore dimension, the wall thickness and the specific surface area, in the range of 2–35 nm, 3–6 nm and 180–250 m^2^ g^−1^, respectively. More importantly, as the products have crystalline walls, the pore properties are resistance to heat up to 700 °C, which may lead to many new applications of mesoporous materials. Moreover, the results demonstrate that the present design concept suggested for titania can be extended to other metal oxide or mixed oxides such as SnO_2_, TiO_2_-ZrO_2_ and TiO_2_-SnO_2_.

## Results and Discussion

**Figure 1:**
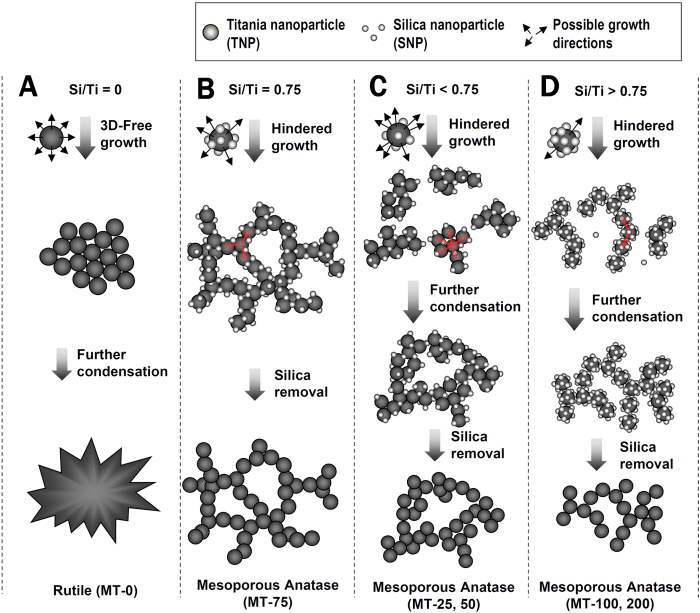
Proposed formation mechanism of mesoporous titania by the action of silica nanoparticles on titania nanoparticles under different Si/Ti ratios: Si/Ti = 0 (**A**), Si/Ti = 0.75 (**B**), 0 < Si/Ti < 0.75 (**C**) and Si/Ti > 0.75 (**D**).

Present synthesis concept is inspired by the principles, which we have found from our previous studies on intercluster salt systems, in which polyoxometalate anions and polycations, both about 1 nm in diameter, are packed alternately into single crystals^51^. The interaction between the cluster ions can be explained as electrostatic (due to the opposite charges) and hydrogen bonding (between the hydrogen donor and acceptor). In the present study (Fig. 1), we used titania nanoparticle (TNP, about 4 nm in diameter) and silica nanoparticle (SNP, about 1.5 nm in diameter). Because of the large difference between the isoelectric points of titania (pH = 5–7) and silica (pH ~ 2), the nature of interaction between TNPs and SNPs can be tuned by the pH. On increasing the pH from < 0 of the initial mixed solution to the pH of about 1.5, SNPs are partially deprotonated while TNPs remain heavily protonated. Under such a condition, there will be attractive interactions between TNP and SNP through hydrogen bonding, forming precipitates. At the same time, because the particle size of TNP is larger than that of SNP, the TNPs can undergo a preferential condensation reaction with TNPs nearby, which are attracted *via* mediation of SNPs, through their uncovered (reactive) surfaces by SNPs. The bound SNPs exert steric hindrance during the condensation of TNPs. As a result, the TNPs form a loose 3D network (Fig. 1B) rather than solid agglomerates (Fig. 1A). Contrast to the post-crystallization of titania after self-assembly with soft templates that may cause structural collapse as mentioned earlier^52^,^53^, the present use of already-crystalline TNP also enables thermodynamically quazi-stable framework in the 3D network, which provides original benefits for the structural sustainability. On hydrothermal treatment of such precipitates, TNPs consolidate into a mesostructured titania framework and further crystallize under confined space induced by SNPs around. Upon removal of SNPs, the nanocomposite becomes a silica free mesoporous titania with fully crystallized framework and well-developed 3D mesopores. On the contrast, when there is no SNP, the same procedure will produce dense crystalline titania. These reaction mechanisms are schematically shown in columns A and B of Fig. 1. In the present work, the Si/Ti ratio is controlled to investigate the performance of SNPs as a new structure directing matter of TNPs for mesoporous titania materials with systematically tuned pore sizes. The mesoporous titania materials, thus synthesized, are denoted as MT-*x* where *x* stands for the percent Si/Ti molar ratio used in the synthesis.

**Figure 2:**
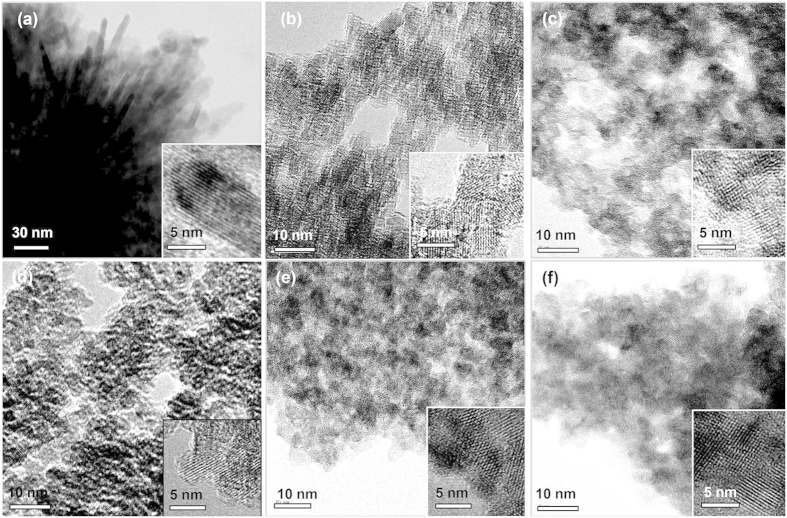
Transmission electron microscopy (TEM) and high resolution TEM (insets) images of MT-0 (**a**), MT-25 (**b**), MT-50 (**c**), MT-75 (**d**), MT-100 (**e**), and MT-200 (**f**).

**Figure 3:**
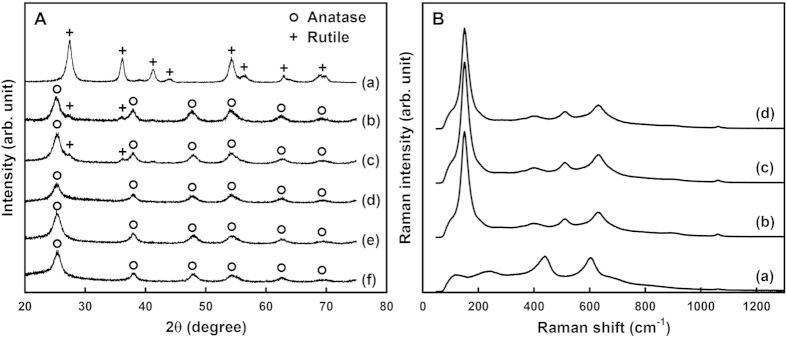
X-ray diffraction patterns (**A**) and Raman spectra (**B**): MT-0 (**a**), MT-25 (**b**), MT-50 (**c**), MT-75 (**d**), MT-100 (**e**), and MT-200 (**f**).

Transmission electron micrographs (TEM) in Fig. 2 reveal the mesostructural evolution of MT-*x* materials as a function of Si/Ti molar ratio (*x*). Urchin-like particles composed of about 10 nm-thick nanorods were obtained in the absence of SNP (MT-0), as shown in Fig. 2(a). To the sharp contrast, the other samples prepared in the presence of SNPs reveal 3D porous networks. The apparent pore size is varied with the *x*-value. The morphological changes from the urchin-like particles to the porous particles are also confirmed by scanning electron micrographs (SEM) (Figure S1). The lattice fringes in the insets of Fig. 2 indicate that all the titania materials exhibit highly crystalline natures of frameworks. The lattice fringe of MT-0 material can be identified as the [101] view of rutile and those of MT-*x* (*x* = 25–200) materials correspond to the [110] views of anatase. The wide angle X-ray diffraction (XRD) patterns and the Raman spectra (Fig. 3) confirmed the phase identification by TEM data of Fig. 2. However, MT-25 and MT-50 materials show rutile phases as a minor component in their XRD patterns. Estimated by the Scherrer’s equation from the half widths of the diffraction peaks, the crystallite sizes are about 9 nm for MT-0 material and about 5 nm for MT-*x* (*x* = 25–200) materials. The blue-shifted E_g_ bands (bulk: 146 cm^−1^ → MT-*x*: 150 cm^−1^) and their peak widths of the Raman spectra of MT-*x* (*x* = 25–200) materials also indicate nanocrystalline anatase frameworks^54^,^55^,^56^,^57^.

**Figure 4:**
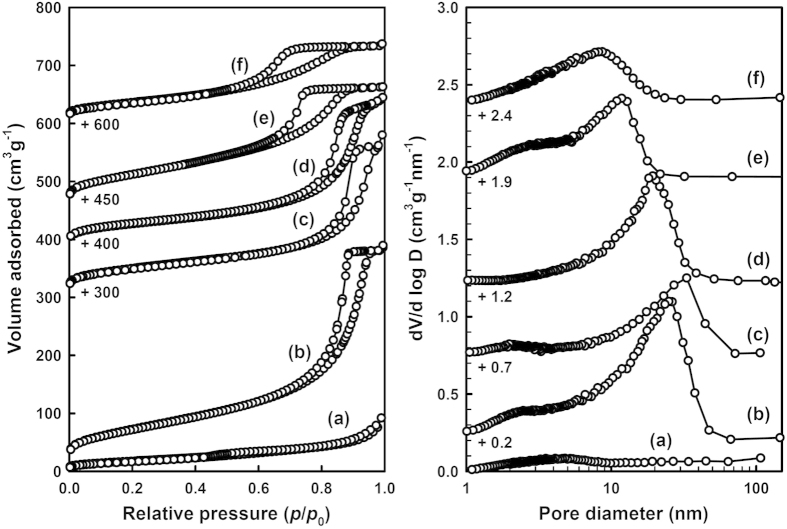
N_2_ adsorption-desorption isotherms (**Left**) and the corresponding BJH pore size distribution curves (**Right**) for MT-0 (**a**), MT-25 (**b**), MT-50 (**c**), MT-75 (**d**), MT-100 (**e**), and MT-200 (**f**).

The mesostructural properties of MT-*x* materials are further evidenced by the N_2_ sorption experiments at liquid N_2_ temperature (Fig. 4). The isotherms of MT-*x* (*x* = 25–200) materials show slight increases in the range of *p*/*p*_0_ = 0.1–0.4 (*p* is partial pressure of adsorbate gas in equilibrium with the surface of material, and *p*_0_ is saturated pressure of adsorbate gas) and steep increases in the range of *p*/*p*_0_ = 0.6–0.95, whereas the MT-0 material shows only a slight increase in the range of *p*/*p*_0_ ~ 0.5. Corresponding pore size distribution curves obtained by the Barrett-Joyner-Halendar (BJH) model^58^ show that the MT-*x* (*x* = 25–200) materials have narrow pore size distributions and their pore sizes span a wide range of 8.5–32 nm, while MT-0 material has only a small amount of mesopores (around 5 nm), which are consistent with the TEM results. The MT-*x* (*x* = 25–200) materials, except of the MT-75, also show small portions of mesopores of 2–3 nm in diameter, which might be present in the titania frameworks. The Brunaur-Emmett-Teller (BET) surface areas of MT-*x* (*x* = 25–200) materials are 180–254 m^2^ g^−1^, considerably larger than 65 m^2^ g^−1^ of MT-0 material. The physicochemical properties of the MT-*x* materials are summarized in Table 1.

The above data clearly substantiate that the present synthesis strategy not only can produce mesoporous titania with a highly crystalline anatase framework but also have a means to control the pore size over a wide range. The variation of the pore size as a function of Si/Ti ratio can be explained with the hindered growth mechanism of TNPs, as shown in Fig. 1, plus other factors depending on the Si/Ti ratio. The repulsive forces between SNPs ensure more or less even distribution of SNPs on the surfaces of TNPs. Therefore, one can expect that the Si/Ti ratio of a reaction mixture is reflected in the fraction of the surface of each TNP that can undergo the condensation into a titania framework in the following hydrothermal reaction. This event, however, is more complicated when the Si/Ti ratio deviates from the optimal value, which appears to occur at Si/Ti = 0.75 (MT-75, Scheme 1B). When the SNP content is smaller than this value (Scheme 1C), the TNPs have chances to bind to other TNPs without intervening SNPs. Small agglomerates of TNPs, surrounded by SNPs will first form and function as the secondary building units that further undergo hindered growth into a 3D porous titania framework. The TNPs in the agglomerated regions will grow in domain size and develop into rutile that can explain the presence of rutile impurity in MT-25 and MT-50 materials as shown in the XRD patterns of Fig. 3. At the same time, occasionally entrapped SNPs inside the TNP agglomerates will eventually form small pores. The size of the TNP agglomerate will be dependent on the Si/Ti ratio. Therefore, the dimension of the large mesopores can be controlled by the Si/Ti ratio. When the Si/Ti ratio is larger than 0.75 (Fig. 1D), the excess SNPs will interfere with the direct interactions between TNPs and, thus, agglomerates between TNPs with SNPs covered on the surface will form. These TNP agglomerates, where the direct TNP-TNP contact is minimized, are different from those in the cases of Si/Ti < 0.75. Further condensation between these agglomerates will form porous titania framework. As the amount of excess SNP increases, the size of such agglomerate will decrease, resulting in smaller pores of the titania framework.

**Figure 5:**
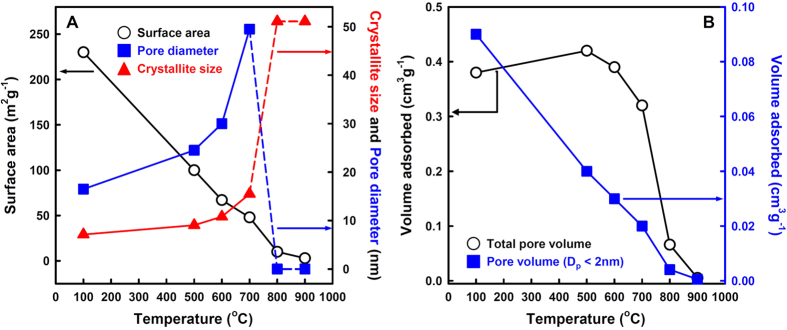
Variations of the properties of MT-100 with heat-treatments at various temperatures (**A**) Surface area (0), pore diameter (▄) and crystallite size (▲), and (**B**) total pore volume (0), and micropore volume (< 2 nm) (▄). The curves for the total surface area and the micropore volume are almost identical except for the scales, indicating that the increase of the crystallite size is due to the coalescence of titania particles around micropores, which does not affect the mesopores. In fact, the coalescence of the titania nanoparticles thins the walls and widens the pores, which is reflected in the slightly increased pore diameter and mesopore volume with the temperature up to 700 °C.

Because the walls are composed of highly crystalline anatase, the MT-*x* materials are expected to have enhanced thermal stability. XRD patterns of MT-100 material depending on the thermal-treatment temperatures are shown in Figure S2. All the peaks in the XRD pattern can be indexed to the anatase phase, which is stable up to 700 °C. At higher temperature, rutile and brookite phases are observed and the anatase phase disappears completely at 900 °C. Crystallite sizes, calculated by using the Scherrer equation, gradually increase with increasing the heating temperatures and are shown in Fig. 5. The changes in surface areas, pore diameters, total pore volumes and micropore volumes of the MT-100 material depending on the heating temperatures are also plotted in Fig. 5. We found that the MT-100 material exhibited excellent thermal stability up to 700 °C, although the crystallite size increases and, hence, the surface area is reduced (Fig. 5).

The simplicity of the synthesis principles allows variation of the synthesis conditions in diverse ways. The size of TNP can be controlled, which can be a means to control the wall thickness of the resultant mesoporous titania. When TNP with a larger size (about 6 nm) was used instead of the TNP with about 4 nm, we obtained thicker walled mesoporous titania (Table 1). Interestingly, the surface area and pore volume of these samples are also larger than those in the MT-*x* materials, indicating that there is a great room to improve the pore properties by varying the reaction conditions. In addition, as metal oxides have considerably different isoelectric points with silica, this method can be extended to various metal oxides other than titania. We have synthesized mesoporous tin oxide by using tin oxide nanoparticles in the place of TNP (Table 1 and Figure S3). Furthermore, this method can be extended to the synthesis of mesoporous materials whose frameworks are composed of mixed metal oxides, TiO_2_-SnO_2_ and TiO_2_-ZrO_2_, materials as shown in Table 1 and Figure S3. The mesoporous mixed metal oxides exhibit homogeneous elemental distribution (Figure S4).

## Conclusion

A facile route for the synthesis of mesoporous crystalline titania by controlling the growth nature of titania nanoparticles using silica nanoparticles is demonstrated. This route produced a series of mesoporous titania materials, which have highly crystalline anatase frameworks, 3D mesoporous structures and high surface areas. The mesopore size could be systematically controlled in a wide mesopore range by simple control of Si/Ti ratio. Moreover, this route could be extended to different sized titania, other metal oxides, or mixed oxide systems. Clearly, the present method will be able to create a vast range of mesoporous materials with crystalline walls, which will provide an opportunity to find many real application fields.

## Experimental Section

### Materials preparation

Aqueous solutions of metal oxide nanoparticles were prepared following the literature methods^59^. For the TNP sol, 11.0 ml of TiCl_4_ (Aldrich, 99.8%) was added drop by drop into the 189 ml of 6 M HCl (Samchun Chemical, 34–36%) aqueous solution under vigorous stirring at 1 °C for 10 min. After aging at room temperature for 3 h and then 16 h at 80 °C, a transparent sol, containing titania nanoparticles with about 4 nm average particle size, was obtained. The tin oxide and mixed oxide nanoparticles are also obtained by a similar method with the preparation of TNP. For the SNP sol solution, 35.0 g of TEOS (Aldrich, 98%) and 35.0 g of ethanol (JT Baker, 98%) were added into 16.0 mL of 10^−3^ M HCl aqueous solution, and further stirred for 12 h at 40 °C. The SNP sol thus obtained was poured into 100 mL of the TNP sol in a designated Si/Ti ratio at 30 °C under vigorous stirring. The mixture solution was titrated by dropwising a 1 M  NaOH solution. After the titration (to reach pH 1.5) and stirring for 1 d at 30 °C, the reaction batch was transferred to a 100 °C oven and kept there for 1 d. White powders were obtained by filtration and drying at 80 °C, which, upon treating in 100 ml of 1 M NaOH aqueous solution at 70 °C for 1 h followed by filtration, produced the MT-*x* samples.

### Materials characterization

X-ray diffraction patterns were obtained from a Rigaku D/MAX 2200 ultima equipped with Cu *K*_α_ at 30 kV and 40 mA. Scanning electron microscope (SEM) images were taken by LEO Supra 55 field emission scanning microscope operating at an accelerating voltage of 15 kV. Transmission electron microscope (TEM) images were obtained using a JEOL JEM-2100F at an accelerating voltage of 200 kV. N_2_ adsorption-desorption isotherms were collected on a Micromeritics ASAP 2000 at liquid N_2_ temperature.

## Additional Information

**How to cite this article**: Lee, H. I. *et al.* Self-arrangement of nanoparticles toward crystalline metal oxides with high surface areas and tunable 3D mesopores. *Sci. Rep.* **6**, 21496; doi: 10.1038/srep21496 (2016).

## Supplementary Material

Supplementary Information

## Figures and Tables

**Table 1:** Physicochemical properties of mesoporous metal oxides.

materials	pore properties			Wall properties	
	S_BET_^a^ (m^2^/g)	V_tot_ b (cm^3^/g)	D_p_ c (nm)	phase d	thickness e (nm)
titania (d ~ 4 nm)					
Si/Ti = 0	65	014	–	rutile	8.3
0.25	254	0.60	23	anatase (rutile)	5.4
0.50	180	0.44	32	anatase (rutile)	5.1
0.75	195	0.38	20	anatase	4.5
1.0	225	0.33	12	anatase	5.1
2.0	132	0.21	8	anatase	4.8
titania (d ~ 6 nm)					
Si/Ti = 1.0	285	0.62	33	anatase	5.7
2.0	245	0.51	27	anatase	5.9
3.0	272	0.59	30	anatase	5.9
tin oxide Si/Sn = 1.00	80	021	13	cassiterite	11.7
mixed oxides					
M = Ti + 0.05 Sn Si/M = 0.75	193	0.30	11	anatase	5.8
M = Ti + 0.05 Zr Si/M = 0.75	177	0.41	18	anatase	5.8

^a^Surface areas were calculated by BET method.

^b^Total pore volumes of the materials were estimated from N_2_ sorption isotherms at *p/p*_0_ = 0.99.

^c^Mesopore sizes were calculated from the adsorption branches of the N2 sorption isotherms by using BJH method.

^d^Crystalline structure were determined from X-ray diffraction patterns.

^e^The wall thickness was calculated by using the Scherrer equation.
