# Supplementary material for: Self-arrangement of nanoparticles toward crystalline metal oxides with high surface areas and tunable 3D mesopores
Source: Sci Rep. 2016 Feb 19;6:21496. doi: 10.1038/srep21496 (PMC4759599; doi:10.1038/srep21496)
Supplement: Supplementary Information [file srep21496-s1.pdf]

Supporting Information for

Self-Arrangement of Nanoparticles toward Crystalline Metal Oxides with High Surface Areas and Tunable 3D Mesopores

Hyung Ik Lee[1], Yoon Yun Lee[2], Dong-Uk Kang[1], Kirim Lee[1], Young-Uk Kwon[1,2,]*, Ji Man Kim[1,2]*

[1]Department of Chemistry, Sungkyunkwan University, Suwon, 440-746, Republic of Korea

[2]SKKU Advanced Institute of Nanotechnology, Sungkyunkwan University, Suwon, 440-746 Korea

*E-mail: ywkwon@skku.edu (Y.-U. Kwon), jimankim@skku.edu (J. M. Kim)

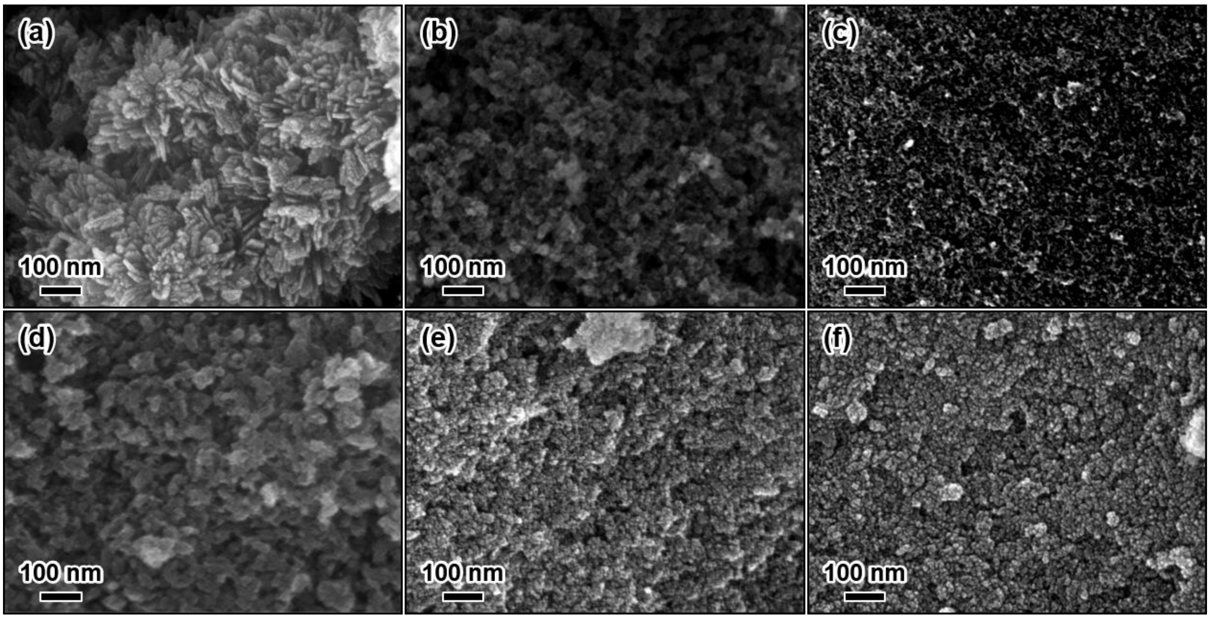

Figure S1. Scanning electron microscopy (SEM) images of MT-0 (a), MT-25 (b), MT-50 (c), MT-75 (d), MT-100 (e), and MT-200 (f).

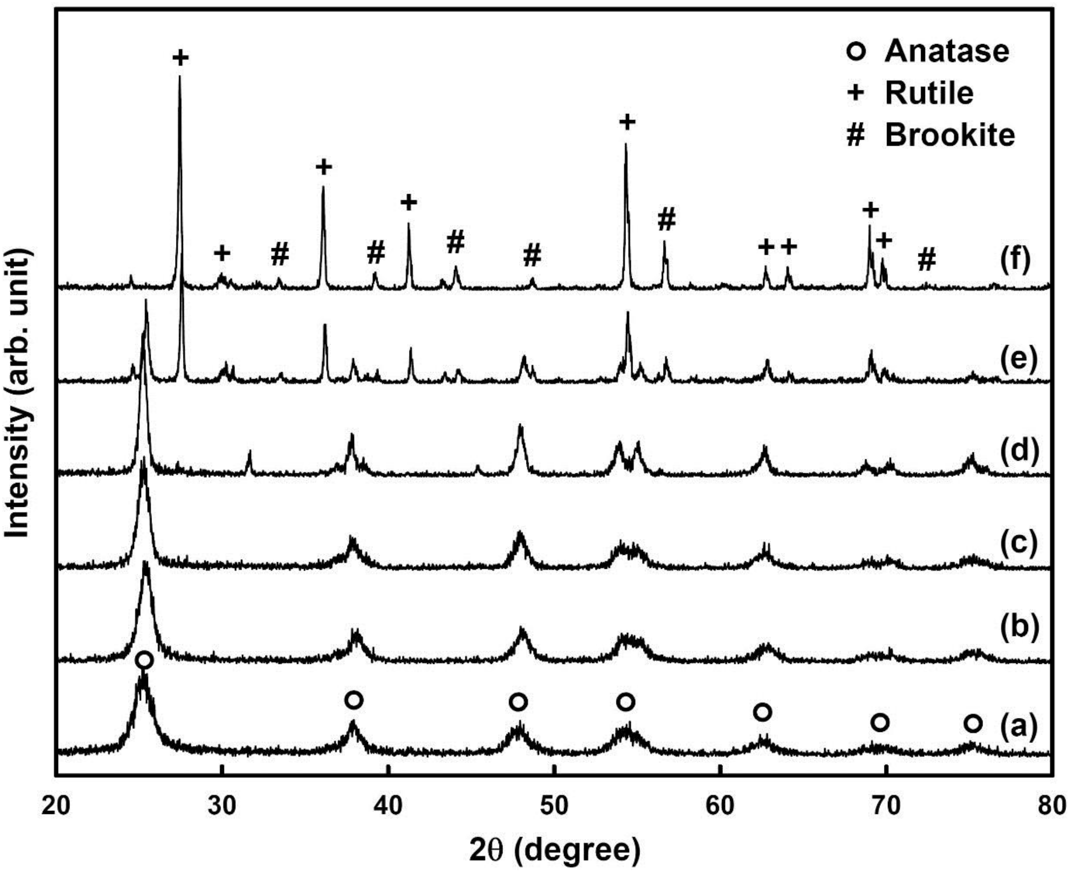

Figure S2. X-ray diffraction patterns of MT-100, before heat-treatment (a) and after heat treatment at 500°C (b), 600°C (c), 700°C (d), 800°C (e), and 900°C (f).

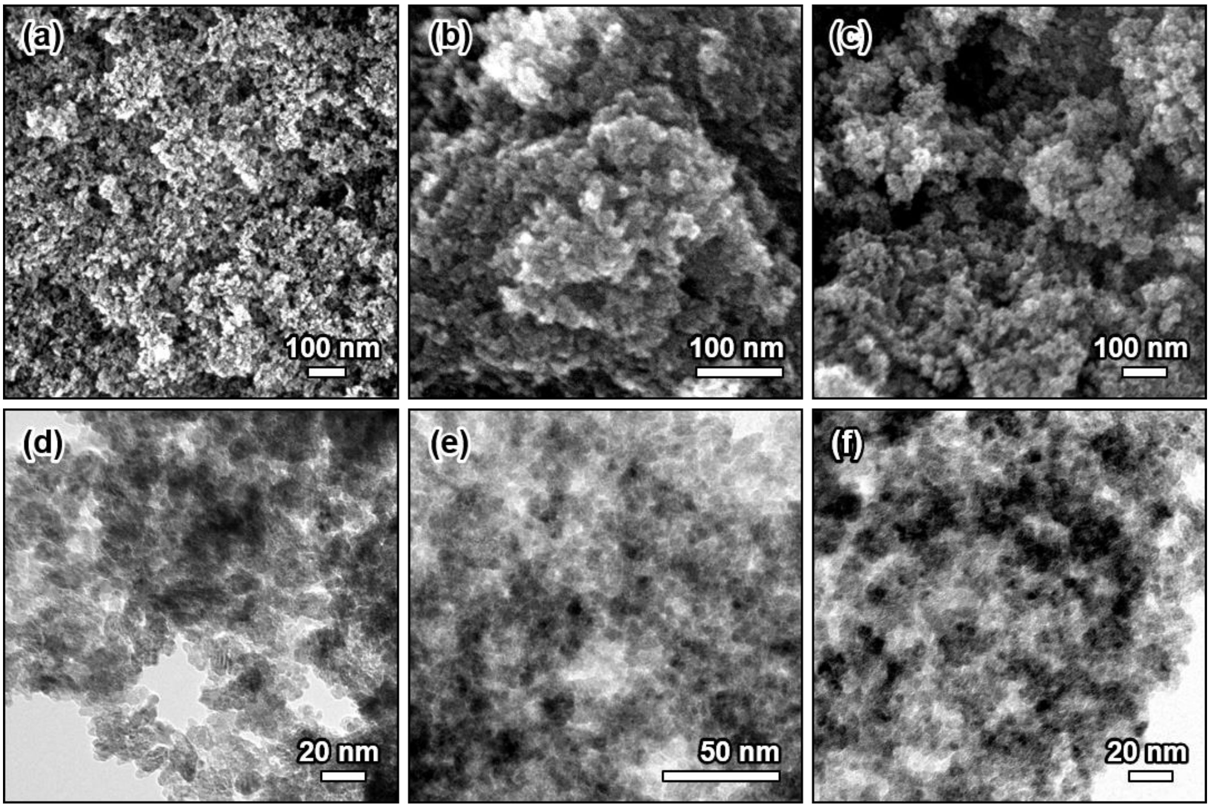

Figure S3. SEM (a-c) and TEM (d-f) images of mesoporous metal oxides composed of various wall materials: The wall materials are SnO_2 (a, d), TiO_2-SnO_2 (b, e), and TiO_2-ZrO_2 (c, f).

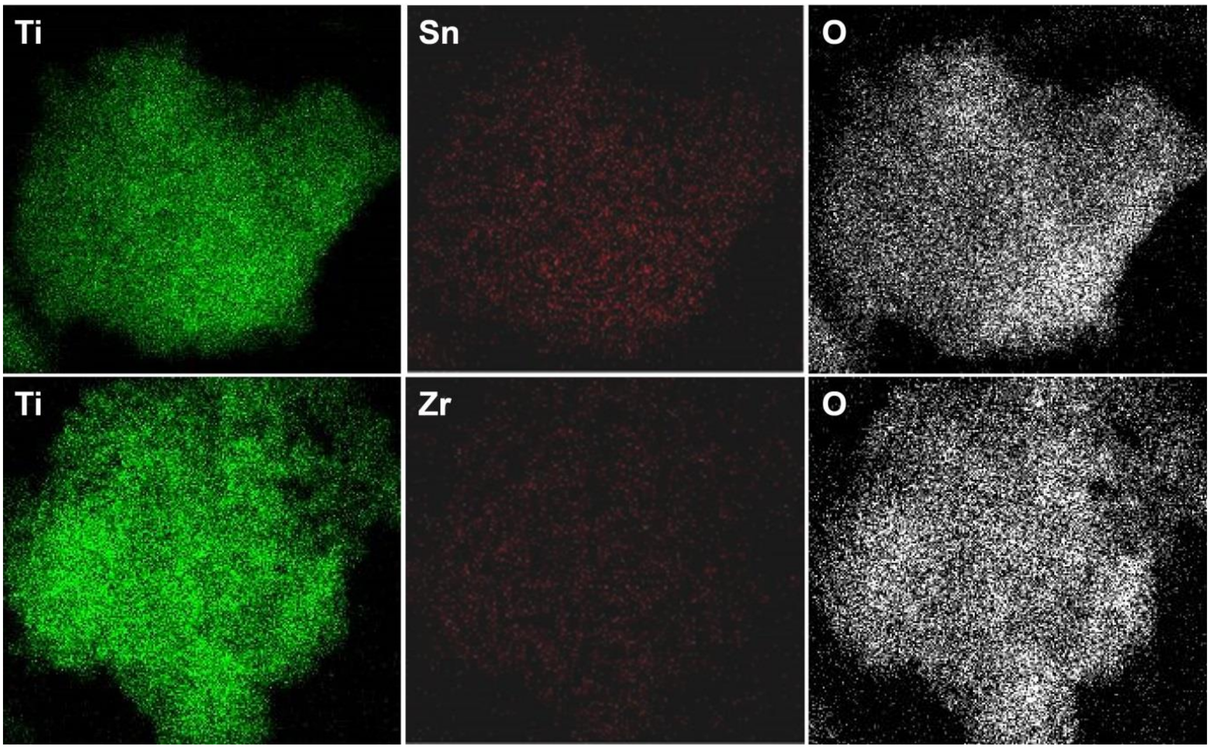

Figure S4. EDS elemental distribution images of mesoporous mixed oxides. Upper three images are on mesoporous TiO_2-SnO_2 and the lower three images are on mesoporous TiO_2-ZrO_2.
